# Intergenerational transmission of parenting and its relation to children's internalizing and externalizing problems in Chinese families

**DOI:** 10.3389/fpsyg.2026.1790080

**Published:** 2026-06-15

**Authors:** Chunli Lu, Zheping Huang, Yiting E

**Affiliations:** 1 School of Normal Education, Longyan University, Longyan, Fujian, China; 2 Department of Pediatrics, Women and Infants Hospital of Rhode Island-Warren Alpert Medical School of Brown University, Rhode Island, RI, United States; 3 School of Marxism, Xi'an Academy of Fine Arts, Xi'an, Shaanxi, China

**Keywords:** children, intergenerational relationships, intergenerational transmission, internalizing and externalizing problems, parenting

## Abstract

**Introduction:**

Although the intergenerational transmission of parenting has been widely documented, its relation to third-generation (G_3_) behavioral problems remains insufficiently explored. Drawing on family systems theory, this study examined how intergenerational relationships mediated the intergenerational transmission of parenting and how this process related to children's behavioral problems.

**Methods:**

We surveyed 2,120 families, including children and their parents, using the Parental Authority Questionnaire, Intergenerational Relationship Scale, and Strengths and Difficulties Questionnaire. Results: (1) G_1_ authoritative parenting was indirectly associated with G_2_ authoritative parenting through intergenerational affection, and was further related to G_3_ internalizing and externalizing problems via the sequential mediation of intergenerational affection and G_2_ authoritative parenting. (2) G_1_ authoritarian parenting was indirectly associated with G_2_ authoritarian parenting through intergenerational conflict, and was further related to G_3_ internalizing and externalizing problems through the sequential mediation of intergenerational conflict and G_2_ authoritarian parenting.

**Discussion:**

By delineating a three-generation transmission mechanism, this study advances understanding of the intergenerational processes through which parenting is associated with child behavioral outcomes. The findings suggest that reinforcing the intergenerational transmission of authoritative parenting and interrupting that of authoritarian parenting may be an effective strategy to reduce behavioral problems in third-generation children.

## 1 Introduction

In recent years, children's behavioral problems have garnered increasing global attention as a critical component of mental health (Liu et al., 2025). Behavioral problems generally refer to maladaptive behaviors that impede social adaptation and are typically categorized into internalizing and externalizing problems (Yang et al., 2022). Internalizing problems involve inwardly directed difficulties such as anxiety and depression, whereas externalizing problems encompass outwardly directed behaviors, including attention-deficit/hyperactivity disorder (ADHD) symptoms, oppositional defiant disorder (ODD) symptoms, and conduct problems (Beauchaine and Zisner, 2017; Yang et al., 2022). These problems are prevalent among school-aged children, especially during the transition from middle childhood to early adolescence (typically spanning grades 3–6) (Eccles, 1999). The childhood-to-adolescence transition is recognized as a time of developmental vulnerability, particularly with regard to students' behavioral problems (e.g., externalizing problems) (Fu et al., 2024). The emergence of internalizing and externalizing behaviors in this stage not only impedes immediate academic performance (Qu et al., 2024) and peer difficulties (Neal and Veenstra, 2021), but may also consolidate into stable maladaptive patterns that persist into adolescence (Fu et al., 2024). Over time, these problems may increase the risk of mental health disorders (Kuijpers et al., 2015) and even contribute to early mortality (Jokela et al., 2009). Therefore, identifying the factors associated with children's behavioral problems remains a key goal in public health.

Among the various determinants of children's internalizing and externalizing problems, parenting has been consistently recognized as a key contextual factor. Extensive studies have demonstrated that parenting substantially affects children's internalizing and externalizing behavioral problems (Alizadeh et al., 2011; Mak et al., 2020; Zhang et al., 2023). However, most of them focus on single-generation parenting, with limited attention to its intergenerational transmission and its associations with child outcomes over time. This limitation constrains our understanding of the mechanisms underlying the intergenerational associations between parenting and developmental outcomes. Furthermore, there remains a lack of empirical evidence regarding how multi-generational processes might be integrated to further optimize intervention strategies for at-risk families. According to family systems theory, families function as interdependent and dynamically interactive systems in which relational patterns, values, and beliefs are sustained across generations (Bowen, 1993). This continuity suggests that the relational climate or behavioral patterns experienced by the second generation (G_2_) within their family of origin serve as the foundational framework for their own transition into parenthood. Specifically, G_2_ observes and internalizes ${\text{G}}_{1^{\text{′}}}$s parenting behaviors—including emotional regulation and conflict resolution styles—which then spill over into the G_2_-G_3_ parental subsystem (Rothenberg et al., 2023). Consequently, this implies that children's behavioral problems are shaped not only by direct parenting but also indirectly through the intergenerational transmission of parenting within the family system.

Existing research has demonstrated the intergenerational transmission of parenting, establishing that both supportive and harsh parenting tend to persist across generations (Campbell and Gilmore, 2007; Chen and Kaplan, 2001; Niu et al., 2018; Rothenberg et al., 2023). However, most of the current mechanism research, largely derived from Western contexts, prioritizes individual psychological traits—such as parental personality or child temperament (Abraham et al., 2022; Capaldi et al., 2003; Shaffer et al., 2009), while overlooking the dynamic relational interactions within the family system. This individual-focused approach fails to fully capture how parenting patterns are functionally sustained and reproduced through systemic processes. Bengtson's Intergenerational Solidarity Model provides a robust theoretical framework for understanding these systemic processes. This model conceptualizes family cohesion through six key dimensions: affectual (emotional closeness), associational (frequency of interaction), consensual (agreement on values), functional (exchange of support), normative (commitment to family obligations), and structural (geographic proximity) (Bengtson and Roberts, 1991). Among these, affectual solidarity refers to the subjective evaluation of the relationship quality between parents and their adult children, which can be measured through positive or negative sentiments, or overall levels of closeness and distance (Bengtson and Schrader, 1982). Although the emotional or intimate quality of the parent-adult child relationship is critical to family dynamics, this construct has remained relatively under-explored in empirical research (Bengtson and Schrader, 1982). In the Chinese cultural context, where family cohesion and intergenerational connectedness are highly valued, the dynamics of parenting transmission may differ substantially. For example, strong emotional bonds between generations may enhance offspring's identification with parental models, thereby reinforcing continuity in parenting practices (Erzinger and Steiger, 2014). Thus, it is necessary to move beyond individual psychological variables to examine intergenerational relational dynamics within the family system, thereby deepening our understanding of how parenting is sustained across generations in China.

Building on this relational perspective, recent studies have examined the effects of G_1_ parenting on G_2_ parenting (Rothenberg, 2019) and the impact of G_2_ parenting on G_3_ behavioral problems (Zhang et al., 2023). Nevertheless, these studies remain largely isolated and lack an intergenerational integration, making it difficult to capture the intergenerational transmission of familial risk and protective factors and how they shape child development. Furthermore, while existing interventions predominantly target single-generation parent-child dyads, there is currently a lack of empirical evidence to support the development of interventions within a broader intergenerational context. This limitation may hinder our ability to fully address the root causes of persistent behavioral patterns. According to the spillover hypothesis of family systems theory, the interaction between grandparents and parents not only shapes parental parenting but may also indirectly affect children's development through these parental practices (Erel and Burman, 1995). Therefore, integrating the parenting and developmental outcomes of all three generations into a unified framework can advance theoretical understanding of intergenerational family mechanisms and inform more comprehensive and sustainable intervention strategies.

In summary, this study aims to examine how G_1_ parenting is transmitted through G_2_ parenting and how these processes are indirectly associated with G_3_ behavioral problems. The findings are expected to provide empirical evidence for the intergenerational transmission of parenting, extend the application of family systems theory within the Chinese context, and offer practical references for developing more comprehensive intervention strategies for children's behavioral problems.

## 2 Literature review and hypothesis

### 2.1 Intergenerational transmission of parenting

Considerable studies have documented the intergenerational transmission of parenting. Early clinical studies, for example, have found a strong association between parents' psychological abuse of their children and their childhood maltreatment experiences (Niu et al., 2018; Simons et al., 1991). Longitudinal evidence further supports this continuity, indicating that individuals exposed to harsh parenting are more likely to reproduce similar practices when raising their children (Rothenberg, 2019). Moreover, studies have also identified the intergenerational transmission of positive parenting. For instance, Chen and Kaplan (2001) found that adolescents who experienced parental warmth and consistent discipline were more likely to demonstrate nurturing parenting in adulthood. While the existence of this transmission is well-documented, its underlying mechanisms and relation to subsequent generations remain insufficiently understood. Existing theoretical frameworks, such as Social Learning Theory and Attachment Theory, have provided foundational explanations for these continuities by emphasizing behavioral modeling and the formation of internal working models (Bandura and Walters, 1977; Bowlby, 1969). However, these mechanisms have predominantly been examined within Western individualistic societies. There remains a gap in understanding how such transmission operates when parenting practices are deeply embedded in collective values and filial obligations. In the Chinese sociocultural context—profoundly shaped by Confucian values that emphasize familial harmony, and intergenerational solidarity—the continuation of parenting across generations may reflect culturally specific dynamics.

### 2.2 The mediating role of intergenerational relationships in the transmission of parenting

Prior research has identified several mediators in the intergenerational transmission of parenting, including G_2_ parents' personality traits, antisocial behaviors, and marital relationships (Capaldi et al., 2003; Caspi and Elder, 1988; Chen and Kaplan, 2001). However, the role of intergenerational relationships remains underexamined. Evidence shows that intergenerational relationships formed during childhood may profoundly shape individuals' later parenting (Beaton and Doherty, 2007), and such relational continuity often extends into adulthood. Even after children have reached adulthood and established independent lives, the intergenerational solidarity between parents and their adult children persists as a powerful force that continues to influence the adult children's own parenting practices (Boszormenyi-Nagy and Ulrich, 1981). Expanding on the multidimensional framework of intergenerational solidarity introduced earlier, this study focuses specifically on affectual solidarity (manifested as intergenerational affection) and its dynamic counterpart, intergenerational conflict, as the primary relational mechanisms. Consistent with the intergenerational solidarity-conflict model, these relationships encompass both affection (i.e., affectual solidarity) and conflict (Bengtson et al., 2002), which collectively define the “relational climate” inherited from one's family of origin. We propose that intergenerational relationships may serve as a psychological bridge for parenting transmission through two primary psychological pathways.

First, intergenerational affection may facilitate the identification and internalization of positive parenting. Social Learning Theory suggests that children are more likely to model behaviors from caregivers with whom they share a warm, secure bond (Bandura and Walters, 1977). When G_1_ parents employ authoritative practices (e.g., warmth and consistent discipline), it fosters high intergenerational solidarity. This positive bond enhances ${\text{G}}_{2}^{\prime }$s trust and emotional security. Consequently, G_2_ parents internalize these nurturing behaviors into their own “internal working models” (Bowlby, 1969), reproducing positive parenting in the next generation. Empirical evidence supports this view, showing that the level of intimacy between fathers and children plays a pivotal role in the transmission of parenting practices. Specifically, G_1_ authoritative parenting fosters a positive intergenerational climate characterized by warmth, trust, and open communication, which in turn promotes the reproduction of similar parenting in G_2_ (Gaunt and Bassi, 2012). Similarly, Hofferth et al. (2012) indicate that individuals who received positive care from their fathers during childhood are more likely to become warm and responsible fathers themselves. Particularly in the context of close father-son relationships, men tend to acquire a sense of parental responsibility by imitating their fathers' behaviors (Guzzo, 2011). Furthermore, existing studies demonstrate that high-quality intergenerational interactions facilitate identification and internalization processes (Erzinger and Steiger, 2014; Grusec et al., 2000), underscoring the mediating role of intergenerational affection.

Second, intergenerational conflict may act as an emotional stressor that perpetuates maladaptive parenting through the “spillover hypothesis.” Family systems theory posits that emotional states generated in one subsystem (G_1_-G_2_) often transfer to another (G_2_-G_3_) (Erel and Burman, 1995). Harsh or neglectful parenting from G_1_ (e.g., authoritarian or permissive styles) often undermines parent-child bonds and intensifies chronic conflict. Such conflict generates negative affect—such as resentment, anxiety, or “hostile attribution bias” (Schori-Eyal et al., 2017) (the tendency to interpret neutral behaviors as intentionally provocative)—which erodes ${\text{G}}_{2}^{\prime }$s emotional regulation. When G_2_ parents face the daily stressors of raising G_3_, this accumulated relational tension may “spill over,” causing them to reflexively revert to the maladaptive patterns they experienced, such as shouting or emotional withdrawal, even if they consciously intended to parent differently. Empirical research indicates that poor mother-child interaction quality leads to increased transmission of parenting styles; specifically, maternal harsh parenting beliefs, whether at high or low levels, are more likely to be inherited by offspring when the relational bond is compromised (Erzinger and Steiger, 2014).

In sum, the intergenerational relationship is not merely a byproduct of 1′s parenting but a dynamic mechanism that either facilitates the transmission of positive practices through emotional closeness or perpetuates maladaptive patterns through the strain of conflict. Based on these theoretical foundations, we hypothesize that intergenerational relationships (affection and conflict) mediate the association between G_1_ and G_2_ parenting.

### 2.3 The mediating mechanisms of intergenerational transmission of parenting and its relation to G_3_ internalizing and externalizing problems

In intergenerational family systems, parenting tends to exhibit continuity across generations. Within the scope of this study, such continuity is conceptualized as a cascading process, whereby G_1_ parenting is associated with G_2_ parenting, which in turn relates to G_3_ mental health outcomes (Rothenberg et al., 2023). While the previous section detailed the psychological mechanisms of parenting transmission, this study extends the literature by examining the developmental consequences of this process for the third generation (G_3_). Family systems theory conceptualizes the family as a set of interconnected subsystems, in which processes within one subsystem (e.g., grandparent–parent) may spill over into others (e.g., parent–child) (Bowen, 1993). In the present study, we focus specifically on this indirect pathway, assuming that G_1_ parenting shapes G_2_ parenting through intergenerational relational processes and that G_2_ parenting subsequently relates to G_3_ internalizing and externalizing problems. Prior research has demonstrated that negative parenting tends to transmit across generations and is associated with adverse mental health in G_3_ children (Kerr and Capaldi, 2019; Rothenberg et al., 2023). Longitudinal evidence further indicates continuity in parenting across generations and its impact on children's internalizing and externalizing problems, with grandparents' caregiving influencing child psychopathology both directly and indirectly through parental practices (Silver et al., 2023). Similarly, G_1_ harsh parenting has been shown to predict G_2_ punitive parenting, which subsequently increases the risk of externalizing behaviors in G_3_ (Bailey et al., 2009). Yet, most existing three-generation studies have been conducted in Western contexts and tend to focus on the behavioral modeling of parenting across generations (Kerr and Capaldi, 2019; Rothenberg et al., 2023). In Chinese families, family relationships are often shaped by Confucian values such as filial piety and intergenerational obligations (Bedford and Yeh, 2019). These cultural orientations may strengthen intergenerational relational bonds and thereby facilitate the internalization or reproduction of parenting models across generations. For example, G_1_ positive parenting may foster intergenerational affection, which facilitates the internalization and continuity of authoritative parenting in G_2_ (Olsen, 1992), thereby reducing G_3_ internalizing and externalizing problems (Zhou et al., 2025). Conversely, G_1_ negative parenting may intensify intergenerational conflict, depriving G_2_ parents of positive caregiving models and increasing the likelihood of reproducing authoritarian or permissive parenting patterns when facing parent–child conflicts, consequently elevating G_3_ behavioral risks (Barnett et al., 2012). Therefore, we assumed that G_1_ parenting is associated with G_2_ parenting through intergenerational relationships, which in turn is related to G_3_ internalizing and externalizing problems.

### 2.4 The current study

Drawing on family systems theory, this study constructed a sequential mediation model to address two primary objectives: (1) to examine the mediating role of intergenerational relationships in the intergenerational transmission of parenting between G_1_ and G_2_ parents; (2) to test the sequential mediation of intergenerational relationships and G_2_ parenting in the relationship between G_1_ parenting and G_3_ internalizing and externalizing problems. Our hypotheses were as follows:

H1: Intergenerational relationships mediate the association between G_1_ and G_2_ parenting.

H1a: G_1_ authoritative parenting is associated with higher intergenerational affection and lower intergenerational conflict, which in turn are associated with higher G_2_ authoritative parenting.

H1b: G_1_ authoritarian parenting is associated with higher intergenerational conflict and lower intergenerational affection, which in turn are associated with higher G_2_ authoritarian parenting.

H1c: G_1_ permissive parenting is associated with higher intergenerational conflict and lower intergenerational affection, which in turn are associated with higher G_2_ permissive parenting.

H2: G_1_ parenting is related to G_3_ internalizing and externalizing problems via the sequential mediation of intergenerational relationships and G_2_ parenting.

H2a: G_1_ authoritative parenting is related to lower G_3_ internalizing and externalizing problems through intergenerational affection and G_2_ authoritative parenting.

H2b: G_1_ authoritarian parenting is related to higher G_3_ internalizing and externalizing problems through intergenerational conflict and G_2_ authoritarian parenting.

**Figure 1:**
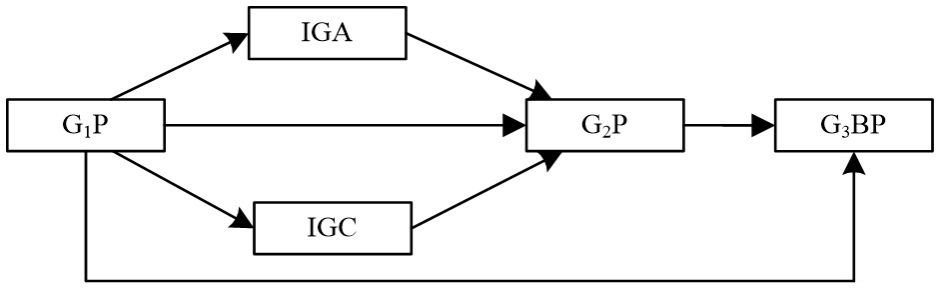
Hypothesis model of the intergenerational transmission of parenting and its relation to G_3_ behavioral problems. G_1_P, G_1_ parenting; G_2_p, G_2_ parenting; G_3_BP, G_3_ behavioral problems; IGA, Intergenerational affection; IGC, Intergenerational conflict.

H2c: G_1_ permissive parenting is related to higher G_3_ internalizing and externalizing problems through intergenerational conflict and G_2_ permissive parenting.

(Figure 1 Hypothesis model of the intergenerational transmission of parenting and its relation to G_3_ internalizing and externalizing problems).

By addressing these aims, this study makes several important contributions. First, it extends research on parenting by empirically examining a three-generation transmission pathway, moving beyond single-generation frameworks. Second, conducted within the Chinese context, this study reveals how family risk and protective factors accumulate and transmit across generations, and provides practical implications for preventing children's behavioral problems within the family context.

## 3 Method

### 3.1 Participants and procedures

Participants were elementary school students and their parents recruited from nine regions in Fujian Province, China (Fuzhou, Quanzhou, Xiamen, Zhangzhou, Longyan, Ningde, Putian, Sanming, and Nanping). Stratified random sampling combined with cluster sampling was employed. Specifically, 2–4 schools were selected from each region, yielding a total of 30 schools. Within each school, 1–2 classes per grade were randomly selected from Grades 3 to 6. Students in grades 1–2 were excluded due to potential limitations in reading comprehension and cognitive capacity for completing self-report questionnaires. In total, 3,500 questionnaires were distributed.

Following ethical approval from the university's ethics committee and consent from participating schools, the research team coordinated with school counselors or head teachers to implement data collection. The final sample included children from Grades 3 to 6 from two-parent households and their primary caregivers (father or mother). After obtaining informed consent, 3,500 elementary students were recruited. Before the survey, school mental health teachers and head teachers received standardized training on instructions, survey procedures, and ethical guidelines. During classroom sessions, questionnaires were administered by teachers following standardized instructions. Students were informed that participation was voluntary, anonymous, and confidential, that they could withdraw at any time without penalty, and that their responses would be used solely for academic research. Each student received a small token of appreciation after completion.

The questionnaire comprised two components: parent and child questionnaires. The parent questionnaire assessed demographic information (e.g., age, education, occupation, and income), both G_1_ and G_2_ parenting, and intergenerational relationships. The child questionnaire measured demographic variables (e.g., gender, grade, etc.) and child outcomes via the Strengths and Difficulties Questionnaire (SDQ). Most children completed the survey during regular class time, which took approximately 30 min.

A total of 3,500 questionnaires were distributed, and 3,200 were returned (response rate = 91.42%). After excluding invalid responses, 3,061 valid student questionnaires remained (validity rate =95.7%), including 1,534 boys (50.11%) and 1,527 girls (49.89%). Grade distribution was as follows: Grade 3 (742, 24.2%), Grade 4 (970, 31.7%), Grade 5 (1,083, 35.4%), and Grade 6 (245, 8.0%), with 21 missing cases (0.7%). Due to graduation exams, Grade 6 participation was relatively low.

During the survey, each child's questionnaire was paired with a parent questionnaire completed by the primary caregiver at home and returned via the head teacher. Participation was voluntary. Of the 3,000 parent questionnaires distributed, 2,650 were returned, and 2,281 were deemed valid after screening. Among respondents, 650 (28.5%) were fathers, 1,629 (71.4%) were mothers, and 2 (0.1%) were missing. Regarding G_2_ educational attainment, 481 (21.1%) had junior high school or below, 555 (24.3%) had secondary vocational, technical secondary school, or high school education, and 1,201(52.7%) had college or higher education (44 were missing, 1.9%). For G_1_ parents, 695 (30.5%) had primary school or below, 905 (39.7%) had junior high school or secondary vocational education, 428 (18.8%) had high school or above, and 253 (11.1%) were missing. Matching child and parent questionnaires via unique ID codes yielded 2,120 valid parent–child pairs. These pairs were distributed across nine regions of Fujian Province, with participant numbers ranging from 79 to 607 due to differences in school sizes and questionnaire validity rates. Region distribution was as follows: Fuzhou (117, 5.52%), Xiamen (607, 28.63%), Quanzhou (106, 5.00%), Zhangzhou (79, 3.73%), Ningde (217, 10.24%), Longyan (559, 26.37%), Putian (110, 5.19%), Sanming (132, 6.23%), Nanping (165, 7.78%), missing datas (28, 1.32%).

### 3.2 Measures

#### 3.2.1 Parental authority questionnaire (PAQ-S) for G_1_ and G_2_ parenting

The PAQ-S was employed to assess parenting styles across two generations, with G_2_ parents serving as the sole informant for these variables. The PAQ-S is an adaptation of the Parental Authority Questionnaire developed by Buri (1991) and revised by Alkharusi et al. (2011). After initial screening and factor analysis from a pilot study, 18 items were retained, covering three dimensions: authoritative (7 items; e.g., “My parents always gave me advice and guidance reasonably and objectively”), Authoritarian (6 items; e.g., “My parents never let me question their decisions”), Permissive (5 items; e.g., “My parents seldom gave me clear expectations or guidance for my behavior”). All items were rated on a 5-point Likert scale (1 = strongly disagree, 5 = strongly agree), with higher scores indicating a greater propensity for the corresponding parenting.

For G_2_ parenting, a self-report format was adopted where G_2_ parents evaluated their own current parenting practices. The items were phrased in the first-person format; for example, the item “My parents always gave me advice and guidance reasonably and objectively” was revised to “I always give my child advice and guidance reasonably and objectively.” In this study, Cronbach's alpha coefficients for authoritative, authoritarian, and permissive subscales were 0.80, 0.75, and 0.63, respectively.

For G_1_ parenting, G_2_ parents used the original third-person scale to report the parenting they received during childhood. Given the practical difficulties in directly surveying the grandparent generation, this study utilized retrospective reports from G_2_ parents regarding their families of origin. Although retrospective measures have inherent limitations, recent empirical evidence suggests that retrospective reports from adult children can accurately reflect the actual parenting they experienced during childhood (Bornstein et al., 2020). Moreover, several studies have demonstrated significant consistency between G_1_ parents' self-reported parenting (prospective data) and G_2_ parents' subsequent retrospective accounts of hostility and rejection (McCullough et al., 2014; Rothenberg et al., 2023; Whitbeck et al., 1992), thereby supporting the validity and feasibility of retrospective measures in intergenerational research. In this study, Cronbach's alpha coefficients for the authoritative, authoritarian, and permissive subscales were 0.75, 0.78, and 0.69, respectively.

#### 3.2.2 Intergenerational relationship questionnaire

The intergenerational relationship questionnaire was developed based on the intergenerational solidarity-conflict theory proposed by Bengtson and Schrader (1982), focusing on the affectual and conflict dimensions to assess G_1_-G_2_ relationships. The affectual dimension assesses positive emotional connectedness between the two generations, including mutual understanding, fairness, trust, respect, and closeness. This subscale comprises 11 items (e.g., “Your parents respect you”; “You respect your parents”), rated on a 5-point Likert scale (1 = strongly disagree, 5 = strongly agree). Higher scores indicate better relationships. The conflict dimension assesses the frequency of interpersonal tensions between the two generations, including arguments, quarrels, criticism, and disagreements. It consists of 3 items (e.g., “You often argue or quarrel with your parents”), which are also rated on a 5-point Likert scale. Higher scores indicate more frequent intergenerational conflict. In this study, Cronbach's α coefficients were 0.92 for the affectual subscale and 0.74 for the conflict subscale.

#### 3.2.3 Strengths and difficulties questionnaire (SDQ)

The Strengths and Difficulties Questionnaire, originally developed by Goodman (2001) and revised by Du Du et al. (2006), was used to assess children's behavior problems. The SDQ comprises 25 items, divided into 5 subscales. In this study, the Emotional Symptoms subscale was used to measure children's internalizing problems, while the Conduct Problems and Hyperactivity subscales were combined to assess externalizing problems. Items were rated on a 3-point Likert scale ranging from 0 to 2 (0 = not applicable, 2 = completely applicable). Higher scores indicate more serious behavioral problems. In this study, Cronbach's α coefficients were 0.72 for the internalizing problems and 0.76 for the externalizing problems.

#### 3.2.4 Covariates

In this study, G_2_ educational attainment, household income, and region were included as covariates. Educational attainment was coded as 1 = junior high school or below, 2 = secondary vocational or high school, and 3 = college or above. Annual household income (in 10,000 RMB) in the past year was coded as 1 = less than 10, 2 = 10–29, 3 = 30–49, and 4 = 50 or more. Region was coded from 1 to 9 based on the 2,023 total GDP rankings of the nine regions in Fujian Province (from highest to lowest): Fuzhou = 1, Quanzhou = 2, Xiamen = 3, Zhangzhou = 4, Ningde = 5, Longyan = 6, Putian = 7, Sanming = 8, Nanping = 9.

### 3.3 Data analysis

SPSS 26.0 and AMOS 21.0 were used for data analysis. First, SPSS 26.0 was used for common method bias analysis, descriptive statistics, and correlation analysis. Second, AMOS 21.0 was used to construct a structural equation model examining intergenerational transmission of parenting and its relation to children's behavioral problems. Third, model fit was evaluated using χ^2^*/df* (< 5), *GFI, RMR, NFI, CFI* (> 0.90), and *RMSEA* (< 0.08) (Hoyle, 2012). Fourth, to generate robust standard errors and 95% confidence intervals (CIs) that account for potential sampling biases and the nested nature of the data, the bootstrap estimation method (5,000 repeated samples) was used. This method was applied not only to provide robust parameter estimates for the structural model but also to test the significance of the mediation effects (Hayes, 2013).

In addition, before constructing the structural equation model, multiple imputation was used to handle the missing data of valid samples (Enders, 2006). Based on the simulation of missing data, 20 new datasets were generated, and regression analyses showed no significant differences across datasets, which were then combined for subsequent analyses.

It should be noted that we examined the potential cluster effect by treating either schools or classes as the clustering unit. We calculated the Intraclass Correlation Coefficient (ICC) for the dependent variables. The results showed that, regardless of whether the analysis was conducted at the school level or the class level, the ICC values were relatively small, approximately 2%, which is well below the conventional threshold of 5% suggested for requiring multilevel modeling (Cohen, 1988). This indicates that the vast majority of the variance resides at the individual level, and the explanatory power of the group level (school/class) is very limited. Consequently, we employed a single-level Structural Equation Model (SEM)—rather than a multilevel approach—to examine the intergenerational transmission of parenting and its impact on the third generation's mental health.

## 4 Results

### 4.1 Control and assessment of common method bias

To minimize common method bias, all questionnaires were administered anonymously and included reverse-scored items. Harman's single-factor test was then conducted. A total of 15 factors with eigenvalues greater than 1 were extracted from the non-rotating exploratory factor analysis, with the first factor accounting for only 15.62% of the variance (< 40%). Therefore, there was no serious common method bias in this study.

### 4.2 Descriptive statistics and correlations

As shown in Table 1, G_1_ authoritative parenting was positively correlated with G_2_ authoritative parenting and intergenerational affection (*p* < 0.01), while negatively related to intergenerational conflict (*p* < 0.01) and G_3_ internalizing and externalizing problems (*p* < 0.05). G_1_ authoritarian parenting was positively associated with G_2_ authoritarian parenting (*p* < 0.01), intergenerational conflict (*p* < 0.01), and G_3_ internalizing and externalizing problems (*p* < 0.05), but negatively associated with intergenerational affection (*p* < 0.01). G_1_ permissive parenting was positively linked to G_2_ permissive parenting (*p* < 0.01), intergenerational conflict (*p* < 0.01), and G_3_ internalizing and externalizing problems (*p* < 0.01), but negatively linked to intergenerational affection (*p* < 0.01). Additionally, both G_2_ educational attainment (*p* < 0.01) and household income (*p* < 0.05) were negatively correlated with G_3_ internalizing and externalizing problems. G_2_ region was positively correlated with G_3_ internalizing (*p* < 0.05) and externalizing problems (*p* < 0.05). Specifically, as regions were coded from 1 (highest GDP) to 9 (lowest GDP), this positive correlation indicates that children in areas with lower economic development exhibit more behavioral problems. Given the large sample size, some statistically significant correlations may reflect small effect sizes.

**Table 1:** Means, standard deviations, and correlation matrix among variables.

*Variables*	*M*	*SD*	1	2	3	4	5	6	7	8	9	10	11	12	13	14
1. GEN	1.72	0.45	1													
2.EDU	6.34	2.61	−0.07^**^	1												
3. AHI	3.41	2.03	−0.01	0.27^**^	1											
4.REG	4.82	2.26	0.04	−0.08^**^	−0.07^**^	1										
5. G_1_Authv	3.47	0.74	0.03	−0.20^**^	0.03	0.00	1									
6. G_1_Autht	2.38	0.81	−0.11^**^	0.04	−0.06^**^	0.02	−0.36^**^	1								
7. G_1_ perm	2.26	0.82	−0.06^*^	−0.03	−0.09^**^	−0.01	−0.39^**^	0.42^**^	1							
8.G_2_ Authv	3.85	0.61	0.06^*^	0.12^**^	0.16^**^	−0.02	0.47^**^	−0.17^**^	−0.25^**^	1						
9.G_2_ Autht	2.18	0.77	−0.09^**^	−0.03	−0.10^**^	−0.02	−0.15^**^	0.44^**^	0.32^**^	−0.27^**^	1					
10. G_2_ perm	1.95	0.79	−0.03	−0.17^**^	−0.13^**^	0.06^**^	−0.10^**^	0.25^**^	0.45^**^	−0.30^**^	0.37^**^	1				
11. IGA	4.21	0.58	0.05^*^	−0.02	0.12^**^	−0.01	0.56^**^	−0.36^**^	−0.35^**^	0.41^**^	−0.23^**^	−0.20^**^	1			
12. IGC	1.81	0.74	−0.07^**^	0.01	−0.09^**^	0.07^**^	−0.24^**^	0.42^**^	0.31^**^	−0.21^**^	0.33^**^	0.32^**^	−0.44^**^	1		
13. G_3_IP	0.57	0.49	−0.02	−0.08^**^	−0.06^*^	0.04^*^	−0.05^*^	0.05^*^	0.07^**^	−0.11^**^	0.11^**^	0.09^**^	−0.04	0.02	1	
14.G_3_EP	0.58	0.33	0.00	−0.08^**^	−0.05^*^	0.06^*^	−0.05^*^	0.05^*^	0.06^**^	−0.14^**^	0.12^**^	0.09^**^	−0.06^**^	0.06^**^	0.57^**^	1

*N* = 2120, ^*^ *p* < 0.05, ^**^ *p* < 0.01. G_2_GEN, G_2_ gender, G_2_ EDU, G_2_ years of educational, G_2_ AHI, G_2_ annual household income, G_2_ REG, G_2_Region, G_1_Authv, G_1_ authoritative, G_1_Autht, G_1_ authoritarian, G_1_ permissive, G_1_Perm, G_2_Authv, G_2_ authoritative, G_2_Autht, G_2_ authoritarian, G_2_ permissive, G_2_Perm, IGA, Intergenerational affection, IGC, Intergenerational conflict, G_3_IP, G_3_ Internalizing Problems, G_3_EP, G_3_ Externalizing Problems.

(Table 1 Means, standard deviations, and correlation matrix among variables).

### 4.3 Sequential mediation of intergenerational relationships and G_2_ parenting in the association between G_1_ parenting and G_3_ internalizing problems

Before conducting the structural equation modeling (SEM) analysis, normality tests were performed on all observed variables. The results showed that the skewness (0.002–0.757) and kurtosis (0.097–0.459) values for each variable fell within acceptable ranges, satisfying the normality assumptions required for maximum likelihood estimation ((Curran et al., 1996)). Subsequently, a structural equation model using maximum likelihood estimation in AMOS 21.0 was conducted to test the sequential mediating roles of intergenerational relationships (affection and conflict) and G_2_ parenting in the association between G_1_ parenting and G_3_ internalizing problems. After controlling for G_2_ educational attainment, household income, and region, the model demonstrated an acceptable fit: χ^2^/df = 10.380 (χ^2^/df tends to increase when the sample size is large), *RMSEA* = 0.067, *GFI* = 0.982, *RMR* = 0.026, *NFI* = 0.957, *CFI* = 0.960. To further examine the potential impact of the nested data structure (students within nine regions) and account for potential clustering effects, we conducted a sensitivity analysis. This involved comparing our primary structural model with an augmented model that included Region as an additional covariate. The results indicated that the inclusion of region did not substantively alter the magnitude or significance of the hypothesized paths (Δχ^2^ = 11.839, Δdf = 8, *p* > 0.05), further supporting the stability and robustness of our intergenerational model.

**Figure 2:**
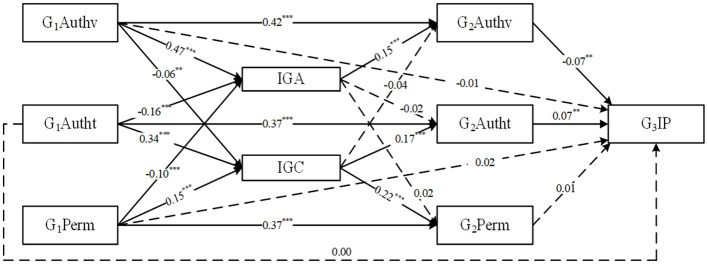
A sequential mediation model of the intergenerational transmission of parenting and its relation to G_3_ internalizing problems. *N*= 2120, ^*^P < 0.01, ^**^P < 0.001. G_1_Authv, G_1_ authoritative; G_1_Autht, G_1_ authoritarian; G_1_Perm, G_1_ permissive; G_2_Authv, G_2_ authoritative; G_2_Autht, G_2_ authoritarian; G_2_Perm, G_2_ permissive; IGA, Intergenerational affection; IGC, Intergenerational conflict; G_3_IP, G_3_ internalizing problems.

Most direct paths were significant. As shown in Figure 2, G_1_ authoritative parenting was directly associated with G_2_ authoritative parenting (β = 0.42, *p* < 0.001) and intergenerational affection (β = 0.47, *p* < 0.001), and negatively with intergenerational conflict (β= −0.06, *p* < 0.01). However, its direct association with G_3_ internalizing problems was nonsignificant (*p* > 0.05). Similarly, G_1_ authoritarian parenting was positively related to G_2_ authoritarian parenting (β = 0.37, *p* < 0.001) and intergenerational conflict (β = 0.34, *p* < 0.001), while negatively related to intergenerational affection (β = −0.16, *p* < 0.001). Its direct association with G_3_ internalizing problems was also nonsignificant (*p* > 0.05). G_1_ permissive parenting was positively associated with G_2_ permissive parenting (β = 0.37, *p* < 0.001) and intergenerational conflict (β = 0.15, *p* < 0.001), and negatively with intergenerational affection (β = −0.10, *p* < 0.001). Its direct association with G_3_ internalizing problems was also nonsignificant (*p* > 0.05). At the G_2_-G_3_ level, G_2_ authoritative parenting was negatively associated with G_3_ internalizing problems (β = −0.07, *P* < 0.01), whereas G_2_ authoritarian parenting showed a positive association (β = 0.07, *p* < 0.01). Intergenerational affection facilitated G_2_ authoritative parenting (β = 0.15, *p* < 0.001), while intergenerational conflict was positively linked with G_2_ authoritarian (β = 0.17, *p* < 0.001) and permissive parenting (β = 0.22, *p* < 0.001).

Mediation analysis further revealed that G_1_ authoritative parenting was indirectly associated with G_2_ authoritative parenting via intergenerational affection [indirect effect = 0.07, 95% CI (0.05, 0.10)]; The total effect was 0.49, and the ratio of the indirect effect to the total effect was 14.29%, partially supporting H1a. G_1_ authoritative parenting was also indirectly associated with G_3_ internalizing problems through a sequential mediation of intergenerational affection and G_2_ authoritative parenting [sequential mediation effect = −0.01, 95% CI = (−0.02, −0.01)]. The total effect was −0.04; the ratio of the sequential mediation effect to the total effect was 25.00%, supporting H2a. G_1_ authoritarian parenting was indirectly associated with G_2_ authoritarian parenting via intergenerational conflict, with a mediating effect of 0.06 [95% CI = (0.05, 0.08)]. The total effect was 0.43; the ratio of the indirect effect to the total effect was 13.95%, partially supporting H1b. Moreover, it was indirectly associated with G_3_ internalizing problems through a sequential mediation involving intergenerational conflict and G_2_ authoritarian parenting [sequential mediating effect = 0.004, 95% CI = (0.001, 0.006)]. The total effect was 0.030; the ratio of the sequential mediation effect to the total effect was 13.33%, supporting H2b. G_1_ permissive parenting was indirectly associated with G_2_ permissive parenting through intergenerational conflict, with a mediating effect of 0.03 [95% CI = (0.03, 0.07)]. The total effect was 0.40, the ratio of the indirect effect to the total effect was 7.50%, partially supporting H1c. However, the sequential mediation pathway from G_1_ permissive parenting to G_3_ internalizing problems via intergenerational conflict and G_2_ permissive parenting was non-significant, indicating that intergenerational relationships and G_2_ parenting fully mediate the association between G_1_ parenting and G_3_ internalizing problems. Overall, these findings indicate that the association between G_1_ parenting and G_3_ internalizing problems operates primarily through indirect pathways involving intergenerational relationships and G_2_ parenting.

(Table 2 Testing the sequential mediating effects of intergenerational relationship and G_2_ parenting on G_1_ parenting and G_3_ internalizing and externalizing problems).

**Table 2:** Testing the sequential mediating effects of intergenerational relationship and G_2_ parenting on G_1_ parenting and G_3_ internalizing and externalizing problems.

Behavioral problems	The path	Mediating effect	*LLCI*	*ULCI*
G_3_IP	G_1_Authv → IGA → G_2_Authv	0.08	0.05	0.10
	G_1_Autht → IGC → G_2_Autht	0.06	0.05	0.08
	G_1_Perm → IGC → G_2_Perm	0.03	0.03	0.07
	G_1_Authv → IGA → G_2_Authv → G_3_IP	−0.01	−0.02	−0.01
	G_1_Autht → IGC → G_2_Autht → G_3_IP	0.004	0.001	0.006
	G_1_ Perm → IGC → G_2_ Perm → G_3_IP	0.001	−0.001	0.001
G_3_EP	G_1_Authv → IGA → G_2_Authv	0.08	0.05	0.10
	G_1_Autht → IGC → G_2_Autht	0.06	0.05	0.08
	G_1_Perm → IGC → G_2_Perm	0.03	0.03	0.07
	G_1_Authv → IGA → G_2_Authv → G_3_EP	−0.01	−0.03	−0.01
	G_1_Autht → IGC → G_2_Autht → G_3_EP	0.005	0.002	0.008
	G_1_ Perm → IGC → G_2_ Perm → G_3_EP	0.001	−0.001	0.001

G_1_Authv, G_1_ authoritative, G_1_Autht, G_1_ authoritarian, G_1_Perm, G_1_permissive, G_2_Authv, G_2_ authoritative, G_2_Autht, G_2_ authoritarian, G_2_Perm, G_2_permissive, IGA, Intergenerational affection, IGC, Intergenerational conflict, G_3_IP, G_3_ Internalizing Problems, G_3_EP, G_3_ Externalizing Problems, *LLCI*, lower limit of the 95% conffdence interval, *ULCI*, upper limit of the 95% conffdence interval.

(Figure 2 A mediation model of the intergenerational transmission of G_1_ and G_2_ parenting and its relation to G_3_ internalizing problems).

### 4.4 Sequential mediation of intergenerational relationships and G_2_ parenting in the association between G_1_ parenting and G_3_ externalizing problems

**Figure 3:**
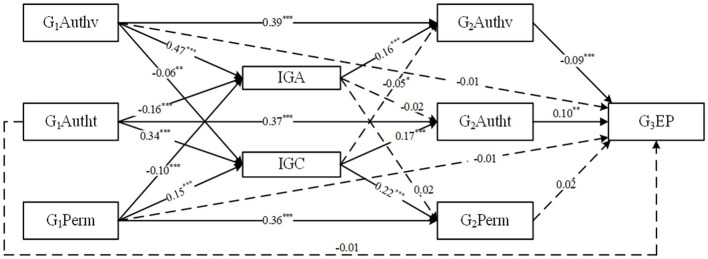
A sequentialed mediation model of the intergenerational transmission of parenting and its relation to G_3_ externalizing problems. *N*= 2120, ^*^P < 0.01, ^**^P < 0.001. G_1_Authv, G_1_ authoritative; G_1_Autht, G_1_ authoritarian; G_1_Perm, G_1_ permissive; G_2_Authv, G_2_ authoritative; G_2_Autht, G_2_ authoritarian; G_2_Perm, G_2_ permissive; IGA, Intergenerational affection; IGC, Intergenerational conflict; G_3_EP, G_3_ externalizing problems.

Similarly, a sequential mediation model was conducted to test the roles of intergenerational relationships and G_2_ parenting in the association between G_1_ parenting and G_3_ externalizing problems. After controlling for G_2_ parental educational attainment, household income, and region, the model demonstrated a good fit: χ^2^/df = 7.727, *RMSEA* = 0.056, *GFI* = 0.986, *RMR* = 0.023, *NFI* = 0.966, *CFI* = 0.970. Most direct paths reached statistical significance. As shown in Figure 3, G_1_ authoritative parenting was positively related to G_2_ authoritative parenting (β = 0.39, *p* < 0.001) and intergenerational affection (β = 0.47, *p* < 0.001). G_1_ authoritarian parenting was positively related to G_2_ authoritarian parenting (β = 0.37, *p* < 0.001) and intergenerational conflict (β = 0.34, *p* < 0.001). G_1_ permissive parenting was positively associated with G_2_ permissive parenting (β = 0.36, *p* < 0.001) and intergenerational conflict (β = 0.15, *p* < 0.001). However, the direct association between G_1_ parenting and G_3_ externalizing problems was nonsignificant (*p* > 0.05). At the G_2_-G_3_ level, G_2_ authoritative parenting was negatively associated with G_3_ externalizing problems (β = −0.09, *p* < 0.001), while G_2_ authoritarian parenting was positively associated with G_3_ externalizing problems (β = 0.10, *p* < 0.01). Intergenerational affection positively predicted G_2_ authoritative parenting (β = 0.16, *p* < 0.001), whereas intergenerational conflict was positively related to both G_2_ authoritarian (β = 0.17, *p* < 0.001) and permissive parenting (β = 0.22, *p* < 0.001).

Sequential mediation analyses indicated that intergenerational affection and G_2_ authoritative parenting fully mediate the relationship between G_1_ authoritative parenting and G_3_ externalizing problems [sequential mediation effect= −0.01, 95% CI (−0.03, −0.01)]. The total effect was −0.05, the ratio of the sequential mediation effect to the total effect was 20.00%, supporting Hypothesis 2a. Similarly, intergenerational conflict and G_2_ authoritarian parenting fully mediate the relationship between G_1_ authoritarian parenting and G_3_ externalizing problems. The sequential mediation effect was 0.006 [95% CI = (0.002, 0.008)]. The total effect was 0.043, the ratio of the sequential mediation effect to the total effect was 13.95%, supporting Hypothesis 2b.

(Figure 3 A mediation model of the intergenerational transmission of G_1_ and G_2_ parenting and its relation to G_3_ externalizing problems).

## 5 Discussion

This study constructed an integrated intergenerational model linking G_1_ parenting, intergenerational relationships, G_2_ parenting, and G_3_ behavioral problems to clarify the mechanisms and outcomes of intergenerational parenting transmission. The results demonstrate that G_1_ parenting is related to G_2_ parenting via two intergenerational mechanisms—intergenerational affection and conflict—which are further associated with G_3_ behavioral development. These findings advance theoretical understanding of parenting continuity and its psychosocial consequences, providing a robust foundation for family intervention targeting children's behavioral development.

### 5.1 The mediating role of intergenerational relationships in the transmission of parenting

The findings indicate that intergenerational affection partially mediates the transmission of authoritative parenting, supporting intergenerational solidarity theory (Bengtson and Roberts, 1991). Specifically, intergenerational affection reflects a positive emotional bond between parents and children, which facilitates the internalization of parental values and identification with parental parenting. When G_1_ parents engage in authoritative parenting characterized by warmth, sensitivity, and consistent regulation, they cultivate a nourishing emotional climate; these stable emotional bonds subsequently increase the likelihood that G_2_ parents will adopt and maintain similar parenting in adulthood (Sears et al., 1957). In the Chinese cultural context, this mechanism may also be understood in light of Confucian family values, which emphasize filial piety, intergenerational responsibility, and family harmony (Bedford and Yeh, 2019). Within this cultural framework, the parent–child relationship is not merely a functional caregiving arrangement but a deeply embedded moral and emotional bond. When G_1_ parents exhibit warmth, consistency, and reasonable norms through authoritative parenting, they fulfill the cultural ideal of “kind parents,” which naturally evokes a sense of gratitude and “filial piety” in the next generation (Chao, 1994). This congruence between parental behavior and cultural expectations provides a normative context that strengthens ${\text{G}}_{2}^{\prime }$s emotional identification with ${\text{G}}_{1}^{\prime }$s practices. Thus, the intergenerational transmission of authoritative parenting in China may reflect not only social learning processes at the individual level but also broader cultural norms that emphasize family harmony and intergenerational responsibility.

The results also indicate that intergenerational conflict partially mediates the transmission of authoritarian parenting, consistent with social learning theory and family systems theory (Bandura and Walters, 1977; Bowen, 1993). It can be explained from three aspects. First, as prior research has noted, poor mother-child interaction quality may exacerbate the intergenerational continuity of maladaptive parenting, because ineffective interaction fails to encourage children to critically evaluate their mothers' parenting. Instead, they may internalize these beliefs uncritically, thereby perpetuating authoritarian patterns (Erzinger and Steiger, 2014). Second, authoritarian parenting, marked by high control and low responsiveness, not only tends to provoke conflict between parents and children, but also reinforces parental authority. In such family contexts, although children may experience negative emotions, they may unconsciously imitate their parents' behaviors. This mechanism is consistent with social learning theory (Bandura and Walters, 1977), which posits that individuals learn behavioral patterns through observation and imitation. When under emotional stress or uncertainty, children are more likely to revert to familiar parenting models, thereby unintentionally reproducing their parents' authoritarian parenting. Third, from the perspective of family systems theory, the family operates as a dynamic system in which individual behavior is shaped by the broader structure of power and normative expectations (Bowen and Kerr, 2009). In authoritarian parenting, intergenerational conflict, though often accompanied by negative emotions, may help maintain the family's hierarchical power structure. This stability may lead children to view authoritarian parenting as an effective method for maintaining order and authority. For example, research has shown that even in families with frequent intergenerational conflict, children are still found to continue to adopt their parents' authoritarian parenting. This is because they have learned how to maintain family authority and power structure through autocratic behavior (Kerr and Stattin, 2000). Thus, intergenerational conflict serves as a key mechanism linking authoritarian parenting across generations.

This research further revealed that intergenerational conflict partially mediates the transmission of permissive parenting. Although permissive parenting is often characterized by low control and limited intervention, conflict is not absent; instead, it may manifest in indirect forms, such as avoidance, emotional withdrawal, or passive tolerance (Baumrind, 1991). Through repeated exposure to these interaction patterns, children may learn that non-intervention and disengagement are acceptable ways to manage tension, rather than engaging in active conflict resolution or boundary setting. Second, permissive parenting is often characterized by a lack of consistent behavioral monitoring and firm boundaries. Research indicates that adolescents under permissive parenting tend to exhibit more intense negative reactions to conflict-provoking situations, suggesting that the absence of clear guidance may impair their emotional regulation and increase the potential for intergenerational friction (Miller et al., 2002), particularly when children seek guidance or support but fail to receive positive responses. As children's developmental needs for structure go unmet, the resulting ambiguity can lead to frustration and friction between generations (Barber, 1996). Over time, children may unconsciously view this emotional detachment and lack of rules as “normal” parental behavior and replicate it in their adulthood to avoid conflict. This intergenerational replication of relational patterns reinforces the continuity of permissive parenting across generations.

### 5.2 Intergenerational transmission of parenting and its relation to G_3_ internalizing problems

The results indicated that G_1_ authoritative parenting was indirectly associated with G_3_ internalizing problems through the sequential mediation of intergenerational affection and G_2_ authoritative parenting. These findings extend previous research by examining three-generation transmission in a Chinese context, addressing the limitation that most studies focused on Western samples (Rothenberg et al., 2023; Silver et al., 2023). First, from the perspective of the spillover hypothesis in family systems theory (Erel and Burman, 1995), G_1_ authoritative parenting may foster warm, supportive, and consistent interaction patterns within the grandparent–parent subsystem. These emotional experiences and relational patterns are likely to “spill over” into the parent–child subsystem, shaping G_2_ parenting practices. Specifically, the warmth, sensitivity, and consistency demonstrated by G_1_ parents in authoritative parenting strengthen intergenerational emotional bonds, making it more likely that G_2_ parents will adopt and maintain authoritative parenting in their own caregiving. Such cross-generational continuity in positive parenting may create a stable emotional climate that supports G_3_ children's emotional adjustment and lowers the likelihood of internalizing problems. Second, from a cumulative protection perspective (Halvorson et al., 2025), the intergenerational transmission of authoritative parenting may generate layered protective effects over time. G_1_ authoritative parenting strengthens intergenerational affection and stabilizes G_2_ parenting, potentially allowing G_3_ children to benefit from sustained emotional support and consistent regulation across generations, thereby buffering against internalizing problems.

The research results also showed that G_1_ authoritarian parenting was indirectly associated with G_3_ internalizing problems through the sequential mediation of intergenerational conflict and G_2_ authoritarian parenting, further extending previous research (Bailey et al., 2009). On one hand, authoritarian parenting is typically characterized by high control and low emotional support (Tung et al., 2019). When this parenting is maintained across generations, G_3_ children have limited opportunities to express emotions, seek support, or develop effective emotion-regulation strategies (Sartaj and Aslam, 2010), thereby increasing their vulnerability to internalizing problems such as anxiety, depression, and withdrawal. On the other hand, our study found that G_1_ authoritarian parenting intensifies intergenerational conflict and fosters emotionally insecure relational patterns in G_2_, which are subsequently reproduced in ${\text{G}}_{2}^{\prime }$s parenting of G_3_. These results provide empirical support for this cumulative process by demonstrating a significant sequential mediation path, suggesting that the adverse impact of 1′s parenting is not only sustained but potentially compounded through the G_2_ family environment, thereby heightening G_3_ children's susceptibility to internalizing problems. Moreover, previous longitudinal research indicates that prolonged exposure to authoritarian parenting may normalize emotional suppression and obedience-oriented interaction patterns within the family system (Lansford et al., 2018). In such contexts, children may learn to inhibit emotional expression to maintain relational stability or avoid conflict, which further reinforces internalizing tendencies. Taken together, these findings suggest that the intergenerational transmission of authoritarian parenting operates as a cumulative risk process in which multiple stressors across generations combine to progressively undermine children's emotional wellbeing (Rothenberg et al., 2023). Therefore, interrupting this cross-generational pattern may be critical for preventing long-term accumulation of internalizing problems and promoting healthier emotional development in future generations.

### 5.3 Intergenerational transmission of parenting and its relation to G_3_ externalizing problems

This study found that the intergenerational transmission of authoritative parenting helps reduce G_3_ externalizing problems. First, G_1_ authoritative parenting fosters robust emotional bonds with their children (Gaunt and Bassi, 2012), which may increase ${\text{G}}_{2}^{\prime }$s willingness to adopt similar parenting. When G_2_ replicates this authoritative parenting, it may provide clear behavioral boundaries and appropriate control, potentially facilitating G_3_ children's internalization of behavioral norms and the development of self-discipline. Second, the transmission of authoritative parenting suggests a potential role in providing G_3_ with a stable, secure, and emotionally supportive environment. In this context, G_1_ authoritative parenting may shape G_2_ through emotional bonds, making G_2_ more likely to continue authoritative practices, thereby maintaining consistent emotional support and behavioral expectations for G_3_. This continuity could potentially strengthen children's self-regulation and sense of responsibility, enabling them to control impulses, manage conflicts effectively, and internalize parental norms (Alizadeh et al., 2011). Consequently, children in such environments are less likely to engage in externalizing behaviors.

This study also found that authoritarian parenting is transmitted across generations via intergenerational conflict, increasing G_3_ children's externalizing problems. The reason may be that authoritarian parenting emphasizes strict behavioral control and obedience with limited emotional support. It often relies on commands, threats, or punishment to shape behavior, which may undermine children's self-esteem, self-efficacy, and emotional regulation. Consequently, children may respond with aggression, defiance, or avoidance (Pinquart, 2017). When G_2_ continues authoritarian parenting, persistent low warmth and high control may create ongoing parent-child tension, potentially forming a cumulative risk environment (Rothenberg et al., 2023). Repeated exposure to emotional suppression and relational stress could hinders norm internalization and self-regulation, making G_3_ children more prone to aggression, rule-breaking, and other externalizing behaviors (Pinquart, 2017). This aligns with the perspective that social and familial determinants of mental health accumulate over time and operate across generations, creating long-term developmental impacts (Yu and Wang, 2026). Our results provide empirical support for the cumulative risk model by demonstrating that the indirect effect of G_1_ parenting on G_3_ is significantly channeled through a cascade of sequential stressors (i.e., intergenerational conflict and subsequent G_2_ maladaptive parenting). Breaking this transgenerational cycle is therefore essential for promoting healthier emotional and behavioral development in future generations.

## 6 Theoretical and practical significance

The result provides both theoretical and practical implications. Theoretically, they confirm that parenting influences extend across three generations, showing how G_1_ parenting is associated with G_3_ behavioral problems via intergenerational relationships and G_2_ parenting. This enriches research on intergenerational transmission of parenting, supports the application of family systems theory in the context of Chinese families, and highlights intergenerational relationships as a key mechanism in family dynamics.

Practically, intervention for at-risk families should consider not only the G_2_-G_3_ relationship but also ${\text{G}}_{2}^{\prime }$s experiences in their family of origin (G_1_). Given that grades 3 to 6 represent a critical precursor to the adolescent transition, interventions during this sensitive period can be particularly effective. First, to break the intergenerational cycle of maladaptive parenting, intervention programmes should be designed to cultivate parents' reflexive capacity. Specifically, practitioners can organize community-based workshops or parental book clubs that encourage parents to enhance self-awareness and critically evaluate their upbringing. These platforms allow parents to identify specific behaviors to retain or modify in a supportive environment. This can be facilitated through structured reflective journaling, group-based guided discussions, or interactive role-playing sessions within schools and communities. Second, the research indicates that positive intergenerational relationships facilitate the transmission of adaptive parenting, whereas intergenerational conflicts mediate the transmission of authoritarian and permissive parenting. Thus, alleviating intergenerational conflicts and strengthening emotional bonds constitute key pathways to reducing the transmission of negative parenting. Family education programmes can incorporate intergenerational emotional development, such as communication training and emotional expression exercises, to foster mutual respect and collaboration between generations. Meanwhile, schools and communities can help families manage conflicts and consolidate cross-generational emotional connections.

## 7 Limitations and future directions

This study has several limitations. First, this study relied on ${\text{G}}_{2}^{\prime }$s retrospective reports of G_1_ parenting and self-report measures for their own parenting (G_2_). This reliance introduces a significant risk of Common Method Bias (CMB). Future research should integrate multi-source data (e.g., G_1_ self-reports, ${\text{G}}_{3}^{\prime }$s perceptions, or behavioral observations) to mitigate such subjective biases and validate the transmission paths. Second, the cross-sectional design limits causal effects; therefore, the observed indirect effects in this study should not be interpreted as true mediation, as establishing such causal mechanisms requires longitudinal research. Future longitudinal approaches are needed to capture the dynamic processes underlying intergenerational transmission and to confirm these mediational pathways over time. Third, the sample was drawn from Fujian Province, China, limiting generalizability to other regions and cultural contexts. Fourth, this study did not fully model the nested structure of the data (e.g., students nested within classes/schools) using multilevel techniques. While the low Intraclass Correlation Coefficient (ICC ≈ 2%) justified the use of a single-level Structural Equation Model (SEM) in the current study, this approach may still overlook potential group-level variances and slightly affect the precision of standard error estimates. Future studies with larger group-level effects should consider formal Multilevel Modeling (MLM) to enhance the precision of estimates. Additionally, the permissive parenting scale showed relatively low reliability (Cronbach's α = 0.63), and the intergenerational conflict scale was brief, potentially restricting measurement breadth and internal consistency. Future research could expand item pools, adopt multi-dimensional or mixed-methods approaches, and recruit more diverse samples across regions and cultures to improve reliability and generalizability.

## Data Availability

The raw data supporting the conclusions of this article will be made available by the authors, without undue reservation.
